# GPR41 and GPR43 in Obesity and Inflammation – Protective or Causative?

**DOI:** 10.3389/fimmu.2016.00028

**Published:** 2016-02-01

**Authors:** Zhiwei Ang, Jeak Ling Ding

**Affiliations:** ^1^Department of Biological Sciences, Faculty of Science, National University of Singapore, Singapore, Singapore

**Keywords:** GPR41, GPR43, gut microbiota, short-chain fatty acids, obesity and inflammation

## Abstract

GPR41 and GPR43 are a pair of mammalian G protein-coupled receptors (GPCRs) expressed in human adipocytes, colon epithelial cells, and peripheral blood mononuclear cells. These receptors are activated by short-chain fatty acids (SCFAs) such as acetate, propionate, and butyrate – which are produced during dietary fiber fermentation by resident gut bacteria. This unique ligand specificity suggests that GPR41 and GPR43 may mediate the interaction between the human host and the gut microbiome. Indeed, studies on knockout mice implicate GPR41 and GPR43 in chronic inflammatory disorders such as obesity, colitis, asthma and arthritis. However, whether GPR41 and GPR43 are protective or causative is inconsistent between studies. This discrepancy may be due to differences in the disease models used, the inbred mouse strains, or non-specific knockout effects. Here, we review the latest findings on GPR41 and GPR43, highlighting contradictory observations. With GPR41 and GPR43 being considered as drug targets, it is pertinent that their role is fully elucidated. We propose that future studies on human tissues, *ex vivo*, may allow us to confirm the role of GPR41 and GPR43 in humans, be it protective or causative.

## Gut Microbiota-Derived Short-Chain Fatty Acids in Metabolism and Immunity

The intestinal microbiota has been linked to a number of beneficial functions: modulating immune development, metabolic function, and preventing diseases such as allergies, colon cancer, and inflammatory bowel disease (1, 2). Some of these effects are at least partly mediated by the short-chain fatty acids (SCFAs), consisting predominantly of acetate, propionate, and butyrate (3), which are produced in millimolar concentrations (around 60, 20, and 20 mM, respectively) in the colonic lumen during the anaerobic fermentation of dietary fiber by saccarolytic gut bacteria (3, 4). In addition to being an important energy source (5), SFCAs have also been shown to affect blood glucose and lipid levels, the colonic environment, and immune functions (6–8).

## Short-Chain Fatty Acids Activate the Mammalian Receptors, GPR41 and GPR43

While the exact mechanisms for the action of SCFAs are still being investigated, a few have been described thus far. Both butyrate and propionate reportedly inhibit histone deacetylases (9–13) while butyrate also activates GPR109A (14). Most notable among the SCFA targets is the mammalian G protein-coupled receptor pair of GPR41 and GPR43, which shares around 42% in aligned peptide sequence identity. The three most abundant SCFAs, namely acetate, propionate, and butyrate, are the most potent agonists for GPR41 and GPR43, with an EC_50_ of around 0.5 mM (15–17). The millimolar concentrations of SCFAs required to activate GPR41 and GPR43 suggest a low potency, especially when compared to other known G protein-coupled receptor (GPCR) ligands such as the chemokine receptor, CCR2, which is activated by the CCL2 chemokine with an EC_50_ of around 1 nM (18). This low potency may restrict the activation of GPR41 and GPR43 to specific locations within the human body, such as in the gut lumen where SCFA concentrations are in the range of 20–60 mM (3). While both GPR41 and GPR43 are activated by SCFA ligands, the downstream G protein coupling specificities differ. GPR41 couples to Gi/G0 protein while GPR43 acts via both Gq/11 and Gi/G0 proteins (15–17).

## GPR41 and GPR43 Expression is Tissue-Specific

The expression of GPR41 and GPR43 has been detected in a variety of tissues; *GPR41* mRNA is detected in adipose tissue, pancreas, spleen, lymph nodes, bone marrow, and peripheral blood mononuclear cells including monocytes (15, 16). GPR41 protein is translated from the bicistronic mRNA encoding *GPR40* and *GPR41*, where an internal ribosome entry site (IRES) is utilized for the *GPR41* coding sequence downstream of *GPR40* (19). *GPR43* mRNA, on the other hand, is found in cells of the distal ileum, colon, and adipose tissue, with the highest expression found in immune cells such as monocytes and neutrophils (15–17). GPR43 expression appears to be modulated during inflammation as immune challenge by lipopolysaccharide (LPS) or tumor necrosis factor (TNF), or treatment with granulocyte-macrophage colony stimulating factor (GM-CSF), was found to raise *GPR43* transcript levels in human monocytes (20, 21). Consistently, luciferase reporter assays have identified inflammation-associated NF-κB (22) and XBP1 (21) transcription factor-binding sites within the GPR43 promoter.

Current data on GPR41 and GPR43 expression is based almost entirely on mRNA measurements, which may not correlate with the expression levels of the functional protein. A few reports do exist on the detection of GPR41 and GPR43 proteins via immunohistochemistry (IHC) with polyclonal antibodies. Through IHC staining, the GPR41 protein is reportedly found in human colon mucosa enterocytes and enteroendocrine cells (23), as well as in mouse autonomic and somatic sensory ganglia (24); while GPR43 has been detected in human and mouse colon epithelial cells (25–27). However, we note that additional controls [such as the use of GPR41/43 knockout mice tissues or multiple antibodies targeting the same receptor but against different epitopes (28)] are required to validate the specificity of the staining. The IHC controls used thus far included a Western blot (25, 26) (which does not demonstrate specificity during IHC since epitope conformations may differ between the two methods) and the absorption test (25, 26) (which may fail to account for non-specific binding by the antigen recognition site). The IHC staining of GPR41 in mouse autonomic and somatic sensory ganglia, as described by Nøhr et al. (24), is perhaps the most convincing as the authors showed colocalization with mRFP under the control of the GPR41 promoter. While the lack of reliable antibodies remains a major challenge toward the further characterization of GPR41 and GPR43, the current data suggest that GPR41 and GPR43 expression is widespread and that these receptors may be involved in a variety of physiological functions.

## GPR41 and GPR43 as Potential Therapeutic Targets for Obesity, Colitis, Asthma, and Arthritis

As receptors specific for SCFAs, the activation of GPR41 and GPR43 may account for some of the physiological effects of the gut microbiome. This is consistent with the findings of some recent knockout mice studies that have implicated GPR41 and GPR43 in the etiology of SCFA-associated chronic inflammatory diseases such as colitis, asthma, and arthritis in mice (Table 1) (29–37). GPR43 has also been associated with diet-induced obesity (34–36, 38–40). These findings have resulted in considerable interest in GPR43 and GPR41 as therapeutic targets (41). In fact, some early synthetic allosteric agonists for GPR43 and GPR41 have already been reported (42, 43).

**Table 1:** **Contradictory findings on the inflammation phenotypes of *Gpr43^−/−^* and *Gpr41^−/−^* mice**.

***Gpr43*^−/−^mice display increased chronic inflammation**	
Maslowski et al. (29)	Exacerbated colitis, arthritis, and asthma
	Reduced neutrophil recruitment
Smith et al. (32)	Exacerbated colitis
	Reduced Treg cell count
Masui et al. (33)	Exacerbated colitis
Macia et al. (44)	Exacerbated colitis
	Reduced IL-18 expression presumably due to reduced inflammasome activation in epithelial cells
***Gpr43*^−/−^ mice display reduced chronic inflammation**	
Sina et al. (30)	Reduced colitis
	Increased neutrophil recruitment
Kim et al. (31)	Reduced colitis
	Reduced ERK and p38 activation in epithelial cells
Vieira et al. (45)	Reduced joint inflammation in mouse model of gout
	Impaired inflammasome formation in macrophages
***Gpr43*^−/−^ mice display increased obesity markers**	
Ge et al. (38)	Increased lipolysis and plasma free fatty acids
Tolhurst et al. (35)	Impaired glucagon-like peptide-1 secretion and glucose tolerance
Kimura et al. (36)	Increased fat accumulation and obesity on a normal diet
McNelis et al. (39)	Glucose intolerance due to defective insulin secretion
	Reduced beta cell mass and expression of differentiation genes
Priyadarshini et al. (40)	Marginal reduction in glucose-stimulated insulin secretion
***Gpr43*^−/−^ mice display reduced obesity markers**	
Bjursell et al. (34)	Improved glucose control and reduced body fat mass on a high-fat diet
***Gpr41*^−/−^ mice display increased inflammation**	
Trompette et al. (37)	Exacerbated asthma
	Impaired dendritic cell generation
***Gpr41*^−/−^ mice display reduced inflammation**	
Kim et al. (31)	Reduced colitis
	Reduced ERK and p38 activation in epithelial cells
***Gpr41*^−/−^*Gpr43*^−/−^ mice display reduced obesity markers**	
Tang et al. (46)	Increased insulin secretion and improved glucose tolerance in type 2 diabetes

## Reports on GPR41 and GPR43 Knockout Mice Phenotypes are Inconsistent

Despite the growing interest in GPR41 and GPR43, many questions regarding their functions remain unanswered. Notably, while knockout mice studies generally agree upon the importance of GPR41 and GPR43 in chronic inflammatory diseases such as colitis, asthma, and arthritis (29–37); the same studies fail to agree on whether GPR41 and GPR43 is protective or causative of these conditions, with both outcomes being reported (Table 1). The inconsistent knockout phenotypes may be due to a variety of factors such as differences in the disease models used, the inbred mouse strains or non-specific knockout effects.

In mouse colitis models, Maslowski et al. (29), Masui et al. (33), and Smith et al. (32) reported that GPR43 knockout increases the severity of colitis; while Sina et al. (30) and Kim et al. (31), on the other hand, conveyed the opposite. The inconsistent knockout phenotypes may be attributable to differences in the protocols used to induced colitis – Maslowski et al. (29) (2.5% DSS for 7 days), Masui et al. (33) (2% DSS for 7 days), Sina et al. (30) (4% DSS for 6 days), Kim et al. (31) (EtOH and TNBS), and Smith et al. (32) (T cell transfer model of colitis). GPR43 knockout is also demonstrated by Maslowski et al. (29) to exacerbate the mouse ovalbumin (OVA)-induced model of asthma while Trompette et al. (37) reported no apparent effect in a house dust mite (HDM)-induced model. Of the two reports on the involvement of GPR41 in inflammation thus far, Trompette et al. (37) described exacerbated asthma in GPR41 knockout mice while Kim et al. (31) described GPR41 knockout mice to show reduced colitis. By contrast, knockout mice studies on GPR43 in energy metabolism appear to have consistent findings. With the exception of one study (34), the remaining five groups reported that GPR43 protects against diet-induced-obesity in mice (35, 36, 38–40).

Questions also persist regarding the cell type and pathways responsible for the effect of GPR43. Bone marrow chimera mice studies from Maslowski et al. (29) and Kim et al. (31) suggest that both marrow-derived cells and non-marrow-derived cells contributed toward the colitis phenotype. However, while Maslowski et al. found that marrow-derived cells play a larger role, Kim et al. reported the opposite. Maslowski et al. (29) suggested that GPR43 signaling reduced immune cell recruitment and expression of inflammatory mediators to attenuate colitis, asthma, and arthritis. Sina et al. (30) proposed that the activation of GPR43 and the downstream p38 mitogen-activated protein kinase in polymorphonuclear leukocytes led to increased cell migration to the colon, exacerbating colitis. Kim et al. (31) described the activation of extracellular signal-regulated kinase 1/2 and p38 mitogen-activated protein kinase signaling pathways in epithelial cells by GPR41 and GPR43 to induce the production of cytokines, exacerbating colitis. The study by Smith et al. (32) described that GPR43 was required for T-cell recruitment to attenuate colitis (which may be due to the fact that a T cell transfer model of colitis was used). Interestingly, Macia et al. (44) suggested that GPR43 was required for the induction of IL-18 expression to reduce colitis severity presumably via increased inflammasome activation in epithelial cells while Vieira et al. (45) suggested that GPR43 exacerbated joint inflammation in a mouse model of gout by inducing inflammasome formation in macrophages. Together, these studies suggest that GPR41 and GPR43 may exert both pro- and anti-inflammatory effects, which are dependent on the disease model used. This lack of consensus, coupled with the limitations of the mouse model (which we discuss in detail in the following section), suggest that the consequences of pharmaceutically targeting GPR41 and GPR43 (41) are not fully understood.

## Human and Mouse GPR41 and GPR43 may be Functionally Divergent

Confirming the role of GPR41 and GPR43 in human physiology is necessary as current published findings are based almost entirely on knockout mice models which often fail to fully mimic human diseases. For example, mutations in the retinoblastoma tumor suppressor gene (*RB*) in humans are causative of, as the name suggests, retinoblastoma. On the other hand, *Rb*^+/−^ mice show no increased incidence of retinoblastoma (47). Another notable example is in the null mutation of the dystrophin gene, which reduces the lifespan of individuals with Duchenne muscular dystrophy (DMD) by ~75%; while dystrophin-deficient mice display minimal clinical symptoms and only a ~25% reduction in lifespan (48). More recently, a systematic comparison of human and mouse gene expression patterns during inflammation revealed a poor correlation (49). This may also explain why current mouse models are unable to fully represent human IBD symptoms (50, 51).

Findings from human and mouse cells cultured *ex vivo*, already point to the possibility of a difference in function among species. GPR43 agonists induced the differentiation of mouse (52) but not human (53) adipocytes. Mouse but not human islets secrete insulin upon GPR43 agonist treatment (40). While these *ex vivo* studies suggest a certain level of functional divergence between human and mouse GPR43 signaling, whether these differences would lead to species-specific responses to SCFAs *in vivo* remain unknown. The task of confirming human GPR41 and GPR43 functions *in vivo* is challenging. A possible avenue may be to employ humanized mouse models or to perform studies using primate models, which are expected to more closely resemble human physiology.

## Future Perspectives

Finally, we propose that future studies on human tissues *ex vivo* or in humanized mouse models, may resolve some of these controversies by allowing us to identify the genuine functions of human GPR41 and GPR43, be it pro- or anti-inflammatory. This knowledge will inform current ongoing efforts to develop pharmacological therapies targeting these receptors (41) and may warn of potentially detrimental side effects.

## Author Contributions

Both the authors listed, have made substantial, direct, and intellectual contribution to the work, and approved it for publication.

## Conflict of Interest Statement

The authors declare that the research was conducted in the absence of any commercial or financial relationships that could be construed as a potential conflict of interest.
